# Sixty‐Two‐Year‐Old Male Suffering From Uremic Leontiasis Ossea Caused by Severe Secondary Hyperparathyroidism

**DOI:** 10.1002/jbm4.10038

**Published:** 2018-03-30

**Authors:** Yogendranath Purrunsing, Jingjing Zhang, Ying Cui, Wei Liu, Yi Xu, Xunning Hong, Changying Xing, Xiaoming Zha, Ningning Wang

**Affiliations:** ^1^ Department of Nephrology First Affiliated Hospital with Nanjing Medical University Nanjing Jiangsu Province People's Republic of China; ^2^ Department of Nuclear Medicine First Affiliated Hospital with Nanjing Medical University Nanjing Jiangsu Province People's Republic of China; ^3^ Department of Radiology First Affiliated Hospital with Nanjing Medical University Nanjing Jiangsu Province People's Republic of China; ^4^ Department of General Surgery First Affiliated Hospital with Nanjing Medical University Nanjing Jiangsu Province People's Republic of China

**Keywords:** UREMIC LEONTIASIS OSSEA, VASCULAR CALCIFICATION, SECONDARY HYPERPARATHYROIDISM, CHRONIC KIDNEY DISEASE‐MINERAL AND BONE DISORDERS

## Abstract

Secondary hyperparathyroidism (SHPT) is a long‐term complication of chronic kidney disease–mineral and bone disorder (CKD‐MBD). SHPT is characterized by hyperplasia of the parathyroid glands and abnormal secretion of parathyroid hormones (PTH), calcium and phosphorous metabolic disorders, renal osteodystrophy, vascular and soft tissue calcification, malnutrition, and other multiple system complications, which can seriously affect the quality of life of the patient and increase the risk of cardiovascular disease and mortality rate. Uremic leontiasis ossea (ULO) is a medical condition only rarely encountered clinically. SHPT causes craniofacial bone deformity accompanied by lesions of the nerve, cardiovascular, respiratory, bone, or other systems within the body. The case discussed here is related to severe SHPT. A 62‐year‐old male patient was suffering from leontiasis ossea, pectus excavatum, vascular calcification, spontaneous bone fractures, and lower limb deformities. He was undergoing hemodialysis and given total parathyroidectomy (TPTX) with autotransplantation (AT). We further analyzed the multivariate therapeutic effects of TPTX on this patient in order to provide clinical data for standardized treatment of individuals with CKD‐MBD. © 2018 The Authors *JBMR Plus* published by Wiley Periodicals, Inc. on behalf of American Society for Bone and Mineral Research.

## Introduction

Leontiasis ossea, also known as leontiasis or lion face, is a form of severe bone remodeling that prevails in patients with chronic kidney disease (CKD) and secondary hyperparathyroidism (SHPT), renal osteodystrophy, Paget diseases, and fibrous dysplasia characterized by craniofacial, ribs, long bones, and spine deformations.^1^, ^2^ Virchow in 1864 first described leontiasis in a patient as the thickening of facial bones.^3^ In 1953, Cohen reported uremic leontiasis ossea (ULO) to be correlated with SHPT.^4^ This malady increases the size of maxillary bones, which expand into the skull sinuses, affecting the ocular, auditory, nasal, and oral areas, causing acute visual deterioration, bilateral optic nerve palsy, bilateral deafness, dysphagia, and dyspnea.^5^

Today's better medical facilities have improved hemodialysis methods, and meticulous patient follow‐up has decreased the incidence of leontiasis. The present case, a male patient suffering from ULO, is a rare form of severe bone remodeling due to uncontrolled SHPT underwent total parathyroidectomy (TPTX) with autotransplantation (AT).^6^

## Case

**Figure 1:**
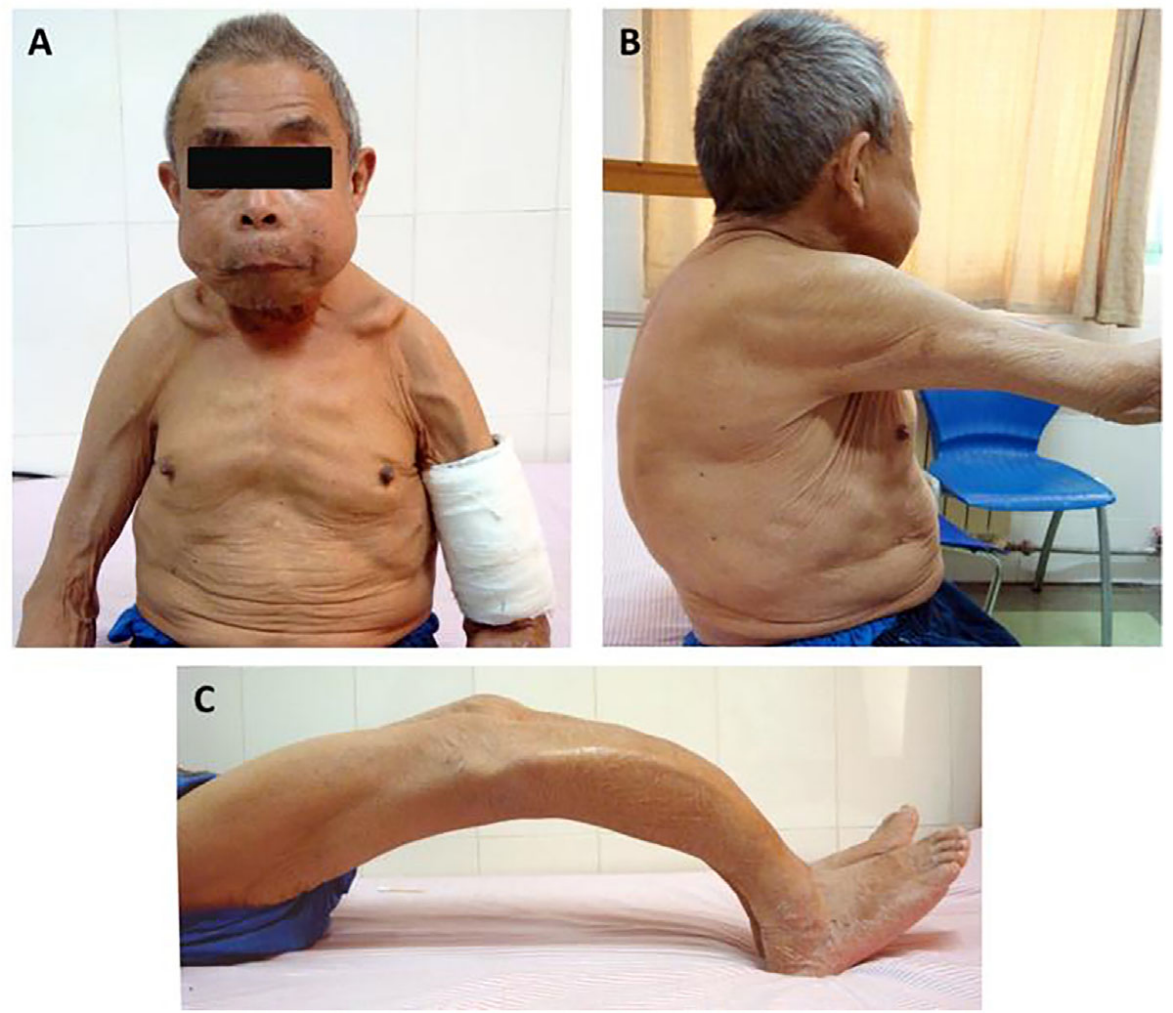
The 62‐year‐old man had obvious facial, thoracic and limb deformities. (A) Craniofacial deformities known as leontiasis ossea and fracture of the left mid shaft of the humerus. (B) The structural deformity of the chest wall is known as pectus carinatum. (C) Lower limbs deformities, the bowing of the legs, consequently lead to his inability to ambulate freely. These pictures were taken before the parathyroidectomy.

A 62‐year‐old male patient diagnosed with chronic glomerulonephritis was maintained on hemodialysis for the previous 12 years, twice per week, with felodipine to control his hypertension. Three years ago, an abrupt surge in his blood serum intact parathyroid hormones (iPTH) levels was observed. One year later, the patient's clinical condition started to deteriorate, showing whole‐body bone deformation and metamorphosis of the lower jaw, chest, and lower limbs. Last year, his serum iPTH was 477 pg/mL, and he was prescribed calcitriol 0.25 µg daily (qd); however, his symptoms continued to be worsen. The patient showed noticeable craniofacial deformities (Fig. 1A), dysphonia, severe bone pain, itching of the skin, inability to walk, and a decrease in body height from 170 cm to 150 cm. The patient mentioned a spontaneous fracture of the left humerus that had occurred 1 year prior though he claimed that he had not had any related accidents. The pretherapeutic blood tests showed severe anemia and hypoalbuminemia, and the patient was given an emergency infusion of red blood cells and albumin. Routine physical examination showed the following: temperature 36.5°C, pulse 66/min, respiration rate 18/min, and blood pressure 140/80 mmHg. Physically, the patient seemed to suffer from malnutrition, lion face/leontiasis (upper mandibular enlargement and deformity), and the oral hard palate showed non‐hardened hyperplasia; there was also severe deformation of the chest known as pectus carinatum (Fig. 1B), kyphosis, and deformity of the lower limbs (Fig. 1C). After admission, routine blood examination showed the following: serum hemoglobin 118 g/L, erythrocyte count 3.33 × 10^12^/L, hematocrit 0.327, serum total protein 59.7 g/L, serum albumin 36.2 g/L, blood urea nitrogen 23.79 mmol/L, creatinine 606.1 µmol/L, serum calcium 2.78 mmol/L, serum phosphorus 1.64 mmol/L, serum iPTH 2183.2 pg/mL, serum alkaline phosphatase (ALP) 1138.7 U/L, serum osteocalcin 244.9 ng/mL, and serum 25‐(OH) vitamin D 37.4 nmol/L.^7^

**Figure 2:**
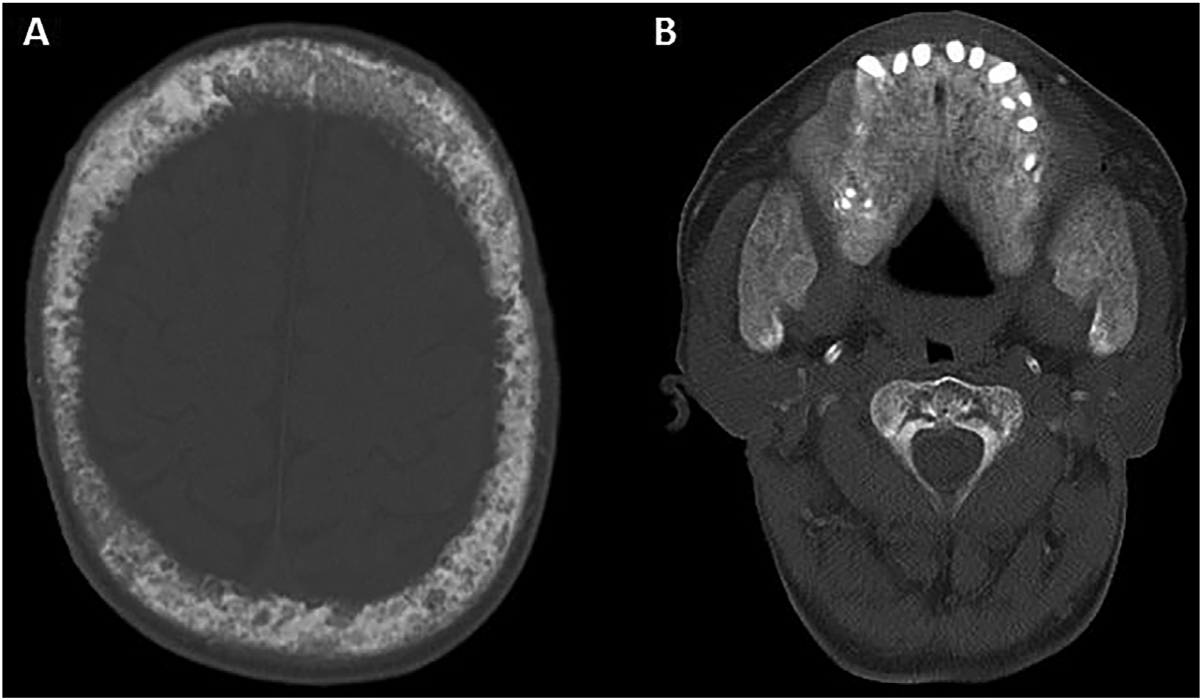
Axial CT of the patient's skull. Thickening was observed in most of the cranial bones, and the maxilla and mandible were profoundly affected. (*A*) There was heterogeneous marked widening of the diploic space of the skull with sclerotic and lytic changes. (*B*) Distinct overgrowths of the ascending rami of mandible with ground glass appearance were seen. The hard palate had been replaced by an enlarged ground glass fibrous structure. CT = computed tomography.

**Figure 3:**
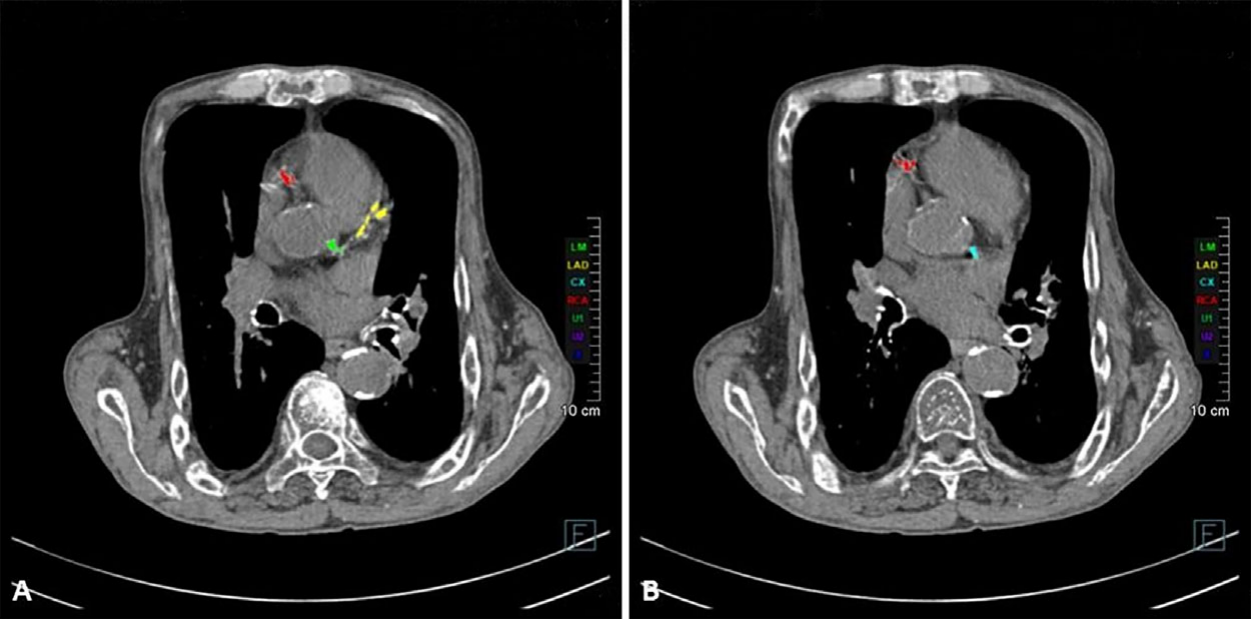
Assessment of coronary calcium score by cardiac CT. The cardiac CT revealed thoracic deformity, vascular and bronchial calcification. (A) Green: left main artery (LMA). Red: the right main artery (RCA). Yellow: left anterior descending artery (LAD). (B) Blue: left circumflex artery (CX).

**Figure 4:**
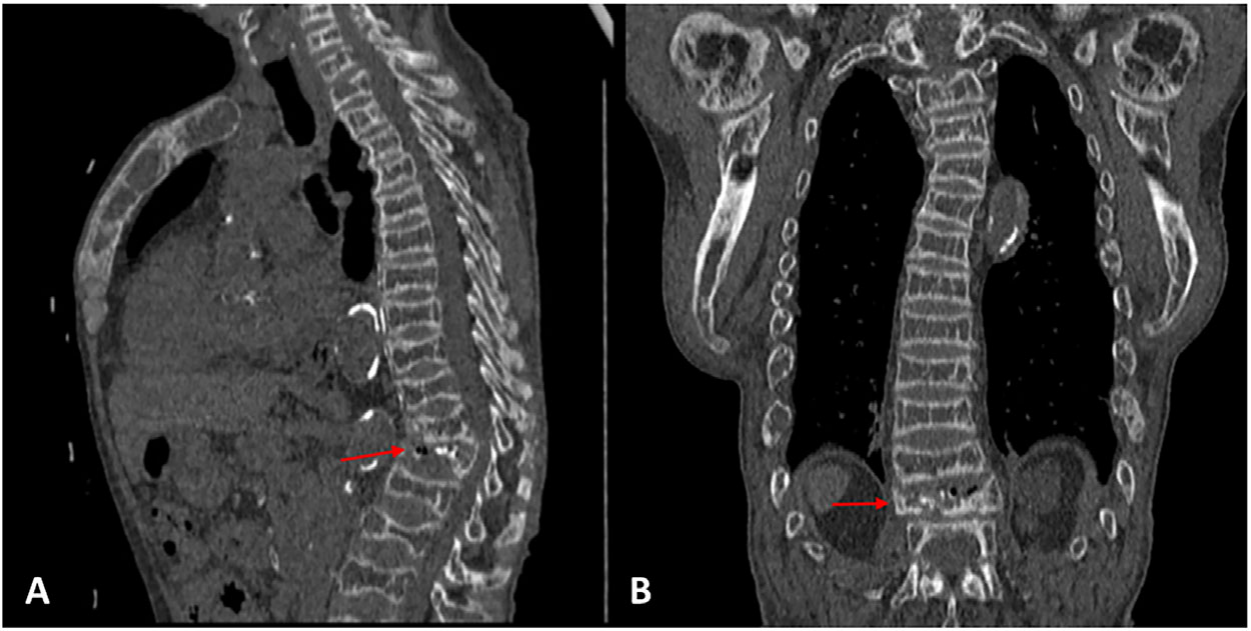
Noncontrast CT scan of the spine. Multiple thoracic and lumbar vertebrae compression fracture and multiple pyramidal instability . (A) Sagittal reformation showed kyphosis and T_12_ wedge deformity (red arrow). (B) Coronal reformation showed T_12_ compression fracture (red arrow).

Auxiliary examinations were as follows: head and cardiac CT scans demonstrated thickening of most cranial bones (Fig. 2A), the maxilla, mandible (Fig. 2B), thoracic deformity, and vascular and heart valve calcification. The Agatston scores (measured in Hounsfield units) of the left main artery (LMA), left anterior descending (LAD) artery, right main artery (RCA) (Fig. 3A) and left circumflex artery (CX) (Fig. 3B) were 163.3, 333.5, 444.1 and 204.2 respectively. The emission computed tomography (ECT) showed hyperparathyroid tissue development (left and right sides, superior and inferior sides of parathyroid glands were detected on the posterior part of the thyroid gland). B‐ultrasound revealed bilateral hypoechoic areas and hyperplasia of the parathyroid. The sagittal and coronal reconstruction images of noncontrast CT showed reduced density of pyramids, multiple thoracic and lumbar vertebral compression fractures (Fig. 4A), multiple pyramidal instability and wedge deformity of T_12_ vertebra (Fig. 4B).

**Figure 5:**
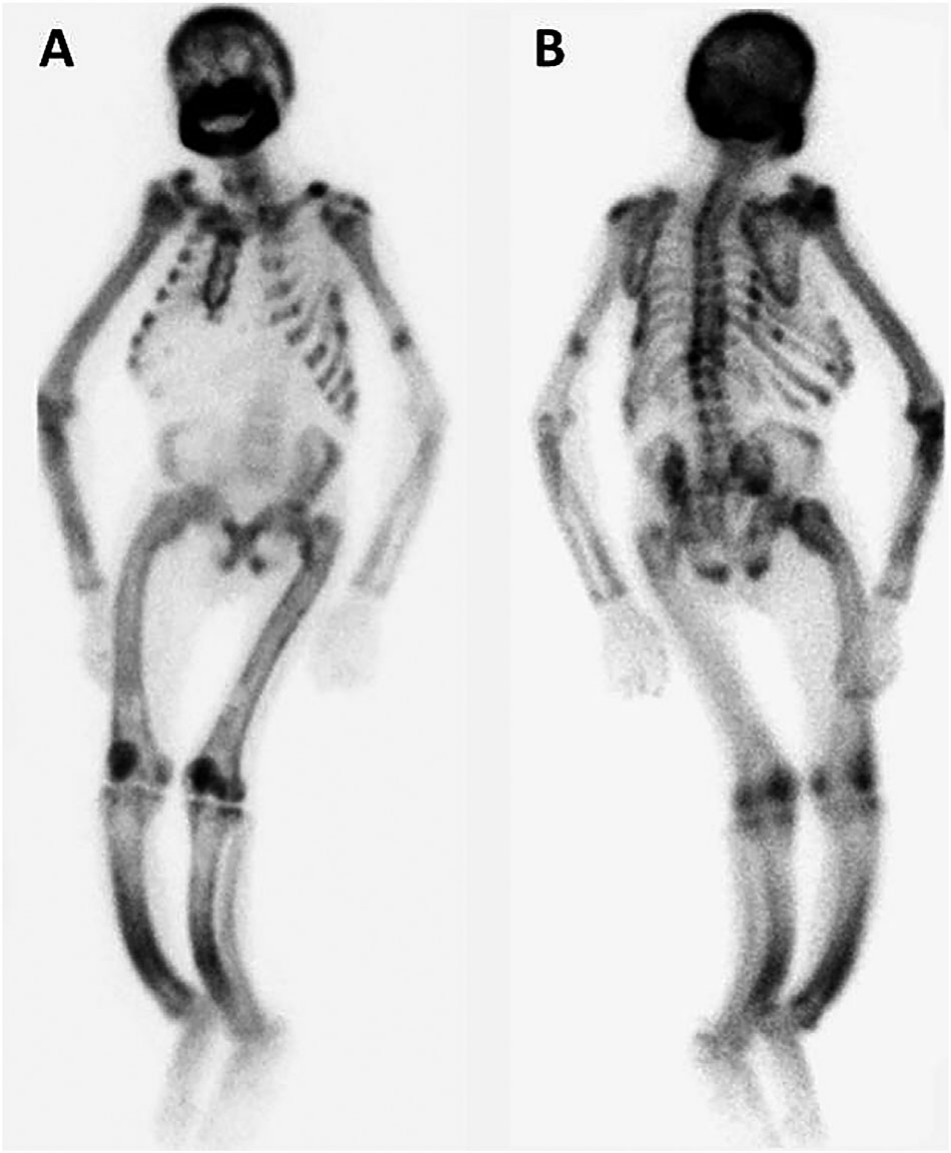
99mTc‐MDP bone scintigraphy, whole‐body scan (A) Anterior view showed bone thickening mainly in the mandible, clavicle, sternum (tie sign) and sacrum. (B) Posterior view showed bone thickening mainly in the scapula, spine and pelvis.

Technetium‐99m‐methylene diphosphonate (99mTc‐MDP) bone scintigraphy indicated an increase in radiotracer uptake especially in the axial skeleton, calvaria, mandible, costochondral junctions, and long bones, and a “tie sign” sternum together with an increased ratio of bone to soft tissue (Fig. 5A). The bone scan showed a higher radionuclide uptake in the left humerus due to prior fracture, spinal kyphosis deformity caused by multiple thoracic and lumbar vertebra compression fracture, and severe bowing of the legs (Fig. 5B). The patient was diagnosed with chronic kidney disease–mineral and bone disorder (CKD‐MBD), SHPT, chronic glomerulonephritis, CKD stage 5, renal anemia, leontiasis ossea, renal osteodystrophy, left humeral fracture, and malnutrition.

**Figure 6:**
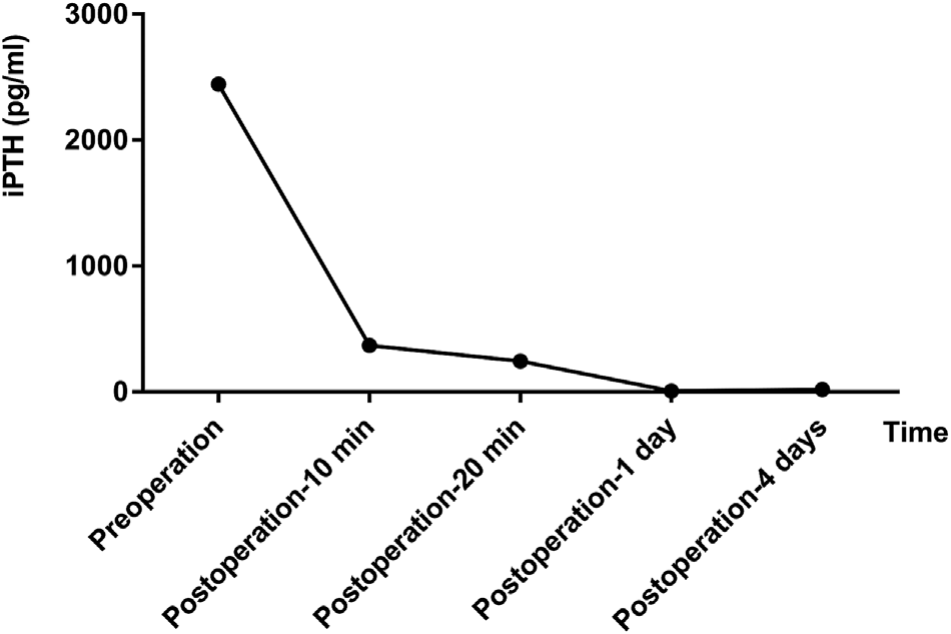
Changes in blood iPTH levels between the preoperative and postoperative periods.

The patient underwent parathyroidectomy in which five glands, including one supernumerary parathyroid gland (SPG) were dissected and removed. The glands weighed 1.9 g, 1.4 g, 1.0 g, 0.2 g, and 0.2 g, respectively.^8^ The pathology report after surgery confirmed all the resected intraoperative frozen sections were parathyroid glands. The forearm without arteriovenous fistula was selected for the site of implantation where the smallest parathyroid gland was sliced into eight pieces (1 × 1 × 1 mm^3^). Venous blood levels of iPTH were determined preoperatively, 10 min, 20 min, 1 day, and 4 days postoperatively, as shown in Fig. 6. Serum iPTH levels were measured using a UniCel DxI800 Access Immunoassy System (Beckman Coulter, Inc., Fullerton, CA, USA).^9^ Vitamin D and calcium (Ca) supplements were prescribed. The patient's clinical condition improved within 10 months of follow‐up, with alleviation of bone pain and cessation of bony overgrowth on the face; however, the deformity of the lower limbs have not yet been corrected, resulting in the inability to walk. His blood pressure returned to normal, without the use of antihypertensive drugs. Blood examination revealed a drop in serum iPTH to 57.2 pg/mL, Ca to 8.8 mg/dL, P to 2.08 mg/dL, and ALP to 297 U/L.^7^

## Discussion

Uncontrolled SHPT causes complications such as bone remodeling, skeletal turnover, cardiovascular disturbances, metabolic imbalances, etc.^10^, ^11^ Tertiary hyperparathyroidism(THPT), first described in 1963, is classically defined as persistent hyperparathyroidism with hypercalcemia due to either prolonged SHPT or autonomous hypersecretion of one or more parathyroid glands after kidney transplantation.^12^, ^13^ Therefore, the term THPT can also be used to describe this patient. In this case, the patient experienced facial and whole‐body remodeling with fracture of the long bone occurring without any traumatic events because of severe SHPT. Henceforth, ineffectual therapeutic methods and insufficient hemodialysis led to prolonged SHPT/THPT. Once the patient was referred to our hospital, we evaluated his case and proposed PTX as according to the Kidney Disease Improving Global Outcomes (KDIGO) guidelines; when a CKD patient with SHPT is experiencing biochemical, radiological, and cardiovascular irregularities and does not respond to medical/pharmacological therapy, parathyroidectomy is recommended.^14^

ULO is the most dramatic pattern of hyperparathyroidism. It involves enlargement of the lower mandible, and poor visualization of the cortical bone due to long‐lasting uremia.^15^ The incidence of vascular calcification in dialysis patients can exceed 80%, with coronary artery calcification, cardiovascular events, and increases in all‐cause mortality.^16^, ^17^ This patient suffered from height shortening of about 20 cm. Uncontrolled SHPT led to renal osteodystrophy, the CT scan (Fig. 4) revealed the compression fractures of the thoracolumbar vertebras and Fig. 1 *C* showed the affected lower limbs, all these combined factors led to the patient's decreased of height. After surgical resection of the parathyroid glands and postoperative follow‐up, bony degeneration ceased but the impairment elicited by the collapse of the vertebras could not be restored.

PTX is recommended for patients with severe SHPT refractory to drug therapy.^18^ Presently, guidelines suggest surgical intervention if severe SHPT is persistently elevated (serum iPTH >800 pg/mL) with hypercalcemia and hyperphosphatemia, and if the case is resistant to drug treatment.^(18)^ This patient suffered from hypercalcemia, so his PTH levels would have been much more higher according to the sigmoidal relationship between Ca and PTH.^19^ Symptoms such as severe ectopic soft tissue calcification, vascular calcification, biopsy revealing osteitis fibrosa cystica or other bone diseases, or ultrasound detecting a single parathyroid volume greater than 500 mm^3^ or diameter greater than 1 cm, also need resection of the parathyroid glands.

Researchers found that individuals who were under hemodialysis for 10 and 20 years had a prevalence of TPTX of 10% and 30%.^20^ Uremic patients often also experience cardiopulmonary insufficiency, vascular calcification, malnutrition, bleeding during the intraoperative and perioperative periods, infection, arrhythmia, circulatory disorders, and other complications.^10^ Improving the safety and effectiveness of TPTX surgery is urgently necessary. Most people have four parathyroid glands; this patient had five, meaning that he had one supernumerary parathyroid gland.^8^ The reported rate of supernumerary parathyroid glands is around 2.5% to 13%.^21^ Studies have shown that SHPT patients undergoing TPTX had an incidence of ectopic parathyroid glands of about 15%, and these glands are mainly distributed around the thymus, esophageal tissue, carotid sheath, and mediastinum.^22^, ^23^ All of these glands must be removed from the neck to ensure the efficacy of the surgery, though ectopic or supernumerary parathyroid glands render the surgery much more difficult. At present, preoperative parathyroid localization tests include ultrasound and ECT. SPECT with 99mTc‐methoxyisobutyl isonitrile as a tracer is also helpful for preoperative localization.

The liver and kidney are the predominant sites of catabolism of blood iPTH, and any dysfunction in either type of the organ can extend the metabolic period of iPTH. Intact PTH has a half‐life of about 2 min in healthy individuals, but in patients with chronic renal failure, iPTH fragments have a degradation time of about 5 min. The current patient's iPTH levels were measured at 10 and 20 min after surgical excision of his hyperparathyroid glands and again on the first and fourth days postoperatively. We proved that for TPTX to be successful, there should be a 82.9% decline in serum iPTH levels after 10 min (sensitivity 85.5% and specificity 73.1%) and 88.9% at 20 min (sensitivity 78.6% and specificity 88.5%).^9^ Blood iPTH >100.5 pg/mL on day 1 (sensitivity 100% and specificity 98.6%) and iPTH >147.4 pg/mL on day 4 (sensitivity 100% and specificity 99.5%) after the operation are considered indicative of persistent SHPT.^9^ This condition should be cautiously managed through appropriate drug treatment and possible reoperation.^9^ In order to avoid this, perioperative blood PTH should be measured regularly to ensure that all parathyroid glands are removed with minimal exploration or injury during the operation.

In conclusion, PTX with autotransplantation is an effective way to treat SHPT caused by chronic renal failure. It relieves patients from mineral bone disorders, alleviates vascular calcification, improves bone health and quality of life. However, abnormal stature caused by vertebral fractures can not be reversed in cases with excessive skeletal malformation.

Hence, CKD‐MBD patients should be educated about their illness, early prevention, multidisciplinary early diagnosis, and treatment plans, thereby improving their quality of life and long‐term survival.^24^ We hope this case report will highlight the awareness of nephrologist across the globe apropos such cases and timely therapeutic measures would be taken.

## Disclosures

All authors state that they have no conflicts of interest.
